# Liquid and Tissue Biopsies for Lung Cancer: Algorithms and Perspectives

**DOI:** 10.3390/cancers16193340

**Published:** 2024-09-29

**Authors:** Paul Hofman

**Affiliations:** 1 IHU RespirERA, Côte d’Azur University, 30 Avenue de la Voie Romaine, 06002 Nice Cedex 01, France; hofman.p@chu-nice.fr; 2 Laboratoire de Pathologie Clinique et Experimentale, Centre Hospitalier Universitaire de Nice, Hospital-Related Biobank (BB-0033-00025), Côte d’Azur University, 30 Avenue de la Voie Romaine, 06002 Nice Cedex 01, France; 3 FHU OncoAge, Pasteur Hospital, Côte d’Azur University, 30 Avenue de la Voie Romaine, 06002 Nice Cedex 01, France

**Keywords:** lung cancer, personalized medicine, molecular biology, liquid biopsy, biomarkers

## Abstract

**Simple Summary:**

A liquid biopsy (LB) is now considered to be a valuable diagnostic tool for advanced and metastatic nonsquamous nonsmall cell lung cancer (NS-NSCLC) patients. The analysis of circulating tumor DNA (ct-DNA) for NS-NSCLC has been shown to increase the chance of identifying the presence of targetable mutations and has been adopted by many clinicians around the world. However, a tissue biopsy (TB) is still the “golden standard” for lung cancer diagnosis and molecular profiling, which is essential for decision-making regarding treatment of NS-NSCLC. Both approaches should be considered as complementary and their sequential or simultaneous use for next-generation sequencing (NGS) can be questionable at tumor diagnosis and/or at tumor progression.

**Abstract:**

The targeted therapies and immunotherapies in thoracic oncology, particularly for NS-NSCLC, are associated with an increase in the number of predictive biomarkers to be assessed in routine clinical practice. These treatments are administered thanks to marketing authorization for use in daily practice or are evaluated during clinical trials. Since the molecular targets to be identified are more and more complex and numerous, it is now mandatory to use NGS. NGS can be developed from both tissue and fluid (mainly blood). The blood tests in oncology, so-called “liquid biopsies” (LB), are performed with plasmatic circulating free DNA (cf-DNA) and are complementary to the molecular testing performed with a TB. LB use in lung cancer is associated with international guidelines, but additional algorithms could be set up. However, even if useful for better care of patients, notably with advanced and metastatic NS-NSCLC, until now LB are not often integrated into daily practice, at least in Europe and notably in France. The purpose of this review is to describe the different opportunities and algorithms leading to the identification of the molecular signature of NS-NSCLC, using both tissue and liquid biopsies, and to introduce the principle limitations but also some perspectives in this field.

## 1. Introduction

**Figure 1:**
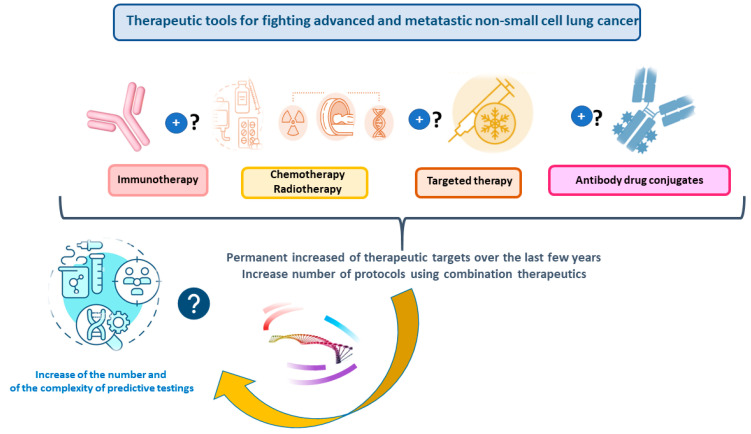
Different therapeutic options for advanced and metastatic nonsquamous nonsmall cell carcinoma and the impact on pathology laboratories.

In these last few years, the therapeutic strategies of thoracic oncology, in particular for NS-NSCLC patients, have improved the overall survival and quality of life of these patients [1]. The different strategies are and probably will be more and more based on different therapies targeting genomic aberrations, but also on different immunotherapies, antibody-drug conjugates, and other new antibodies (Figure 1) [2,3,4,5,6]. Mastering the turnaround time (TAT) to obtain the results of the biomarkers is mandatory for optimal treatment delivery. Moreover, molecular testing has to demonstrate robustness (in terms of sensitivity and specificity), but also cost effectiveness and a widespread availability for efficacy in daily practice. Technological advances (such as new methods for pulmonary endoscopy) and new practices to obtain samples of lung tumors [endobronchial ultrasound (EBUS)- transesophageal ultrasound (EUS)-guided needle aspiration and radial-probe EBUS-guided methods] provide a reliable diagnosis and/or staging of the disease, but also detect smaller lung tumors. However, this has led to sampling of small tissue specimens with a variable percentage of tumor cells that are used for both diagnosis and molecular analysis [7,8,9]. In this context, it is critical to manage efficiently the use of these samples and to master the different algorithms both for immunohistochemistry (IHC) and molecular testing to obtain the mandatory predictive biomarkers of NS-NSCLC [7]. The major challenge is to avoid excessive use of the samples for these tests since this leads to tissue exhaustion and, so, to missing the possibility of having all the results that are required to propose personalized medicine [7,8,9]. In this context, it is necessary to limit the number of tissue sections for diagnosis and prognosis (notably for IHC), but also to reduce multiple and sequential one gene sequencing for predictive biomarker assessment using RT-PCR. Thus, to follow the international recommendation, the ideal approach that gives all the molecular targets is NGS [7,10]. However, NGS with TB can face a number of issues: (i) a longer TAT to obtain the results, (ii) an elevated cost, (iii) a variability in the sensitivity and specificity according to the sequencing technology, the gene panel size, the percentage of tumor cells, and the quality and quantity of extracted nucleic acid from tissue and cytological material, and (iv) a nonavailability of a NGS platform and of expert staff members [7].

The LB of blood has emerged in recent years as a promising approach to identify predictive biomarkers of responsiveness and resistance to targeted therapies as well as to the different tyrosine kinase inhibitors (TKIs) for NS-NSCLC [11,12,13,14]. Therefore, many actionable genomic alterations can be identified from ct-DNA by both one-gene sequencing and NGS approaches [15,16,17,18,19,20,21]. At tumor diagnosis, a TB is certainly the gold standard approach to identify the predictive biomarkers of NS-NSCLC, and LB has to be considered as a complementary possibility to obtain this information. However, different strategies can be proposed where both the TB and the LB or the LB alone in a first setting are performed at tumor diagnosis and/or at tumor progression.

This review addresses different strategies for LB use in NS-NSCLC, and different solutions that are now available. The limitations and some potential algorithms associated with LB use in daily practice today and in the future will be described.

## 2. Liquid Biopsy for Nonsmall Cell Lung Cancer: One Gene Sequencing Testing versus NGS

Initially, the first targeted therapies using TKIs for NSCLC were administered to patients with a mutated *EGFR*, specifically in advanced and metastatic NS-NSCLC with an actionable L858R or del 19 *EGFR* mutation or a resistant T790M *EGFR* mutation. One-gene sequencing test of *EGFR* was developed first from tissue samples, but also from cf-DNA in plasma samples, using RT-PCR or digital PCR (dPCR) methods [15,16,22]. These molecular tests show variable sensitivities and specificities [16,18]. An LB may be requested at tumor diagnosis or at tumor progression for *EGFR* TKI-treated patients [16,18,23]. Therefore, other targeted therapies were developed for NS-NSCLC, notably those targeting *ALK* rearrangements [19]. Specifically, the molecular testing used with an LB for the assessment of *ALK* fusions showed variable sensitivities and specificities at tumor diagnosis [17,20]. The number of molecular targets that can be associated with personalized medicine at tumor diagnosis but also at tumor progression has increased, which has made the use of sequential one-gene sequencing methods from LB more and more difficult [2,4]. In this regard, NGS methods and gene panels were developed from cf-DNA using amplicon-based and hybrid capture sequencing technologies [2,15,24]. Currently, the size of gene panels is variable, and small, medium, and large panels (from 12 to more than 500 genes) can be used with an LB. Table 1 shows a nonexhaustive list of NGS panels for LB use that are currently commercialized or that are in development [2,15]. The choice of one sequencing platform and the panel size can be based on different parameters, including the volume of blood samples to be analyzed each week, the possibility of including patients in clinical trials, the budget and the reimbursement availability, and the expertise of the team, including the bioinformatic capabilities. Each technology presents limitations and advantages. Briefly, the two current technologies used in daily practice for NGS LB are the hybrid capture—and amplicon-based enrichment approaches, and each is associated with advantages and disadvantages. Since hybridization probes can bind to the gene of interest as well as flanking sequences, an important advantage of hybrid capture is certainly that it can identify gene fusions in a partner gene–agnostic manner. This point is pivotal when using NGS LB since the sensitivity of fusion detection from ct-DNA is lower than in TB. Hybrid capture can also recognize DNA sequences that are more divergent from the target sequence compared with amplicon-/PCR-based methods. Therefore, hybridization probes are usually less specific for the target DNA sequence compared with PCR primers in amplicon-based enrichment. The hybrid capture method is also more complex and usually requires a larger nucleic acid sample input compared with amplicon-/PCR-based methods. This is particularly critical when a low amount of ctDNA is available.

Taken together, NGS with an LB holds the advantage of allowing the sequencing of many genes in one step to assess actionable and resistant genomic alterations, which can be drug-targeted [22]. Moreover, these approaches can identify different comutated genes (such as *TP53*, *KEAP1*, and *STK11*), which soon may be taken into consideration to stratify treatment decision-making, in particular in the field of immune-oncology [25,26,27]. However, despite different constraints, one gene sequencing approach is still largely used with an LB, notably for *EGFR* status evaluation, due to the frequency of *EGFR* mutated NS-NSCLC, notably in Asia, and the availability of the different TKIs for treatment of patients with these tumors [28,29,30]. Moreover, in comparison to RT-PCR and dPCR methods, NGS, particularly with an LB, usually needs more extracted ct-DNA, is associated with a longer TAT to obtain the results, and has a higher cost than those associated with one gene sequencing testing.

**Table 1:** Examples of some next-generation sequencing panels currently used with a liquid biopsy.

Company (City, Country)	Gene Panel (REF)	Number of Genes	Sequencing Approach	Sequencing Technology	System	Alternative NGS Compatibility
ThermoFisher Scientific (Waltham, MA, USA)	Oncomine™ Lung cfDNA Assay [31]	11	AmpliSeq	TFS: ion torrent: semiconductor technology	GeneStudio S5	N/A
ThermoFisher Scientific (Waltham, MA, USA)	Oncomine Precision Assay [32]	50	AmpliSeq	TFS: ion torrent: technologie de semi-conducteur	Genexus	N/A
Pillar Biosciences (Natick, MA, USA)	OncoReveal Core and Fusion panel [33]	20	AmpliSeq	Illumina: sequencing by synthesis	NextSeq550Dx	N/A
Hedera Dx (Epalinges, Switzerland)	Hedera Profiling 2 ctDNA panel [34]	32	Hybrid Capture	Illumina: sequencing by synthesis	NextSeq550Dx and Higher	Element Aviti (sequencing-by-binding chemistry
Agilent (Santa Clara, CA, USA)	Beta test; Avida methyl 3400 DMR cancer [35,36]	169	Hybrid Capture	Illumina: sequencing by synthesis	NextSeq550Dx	Element Aviti (sequencing-by-binding chemistry)
Illumina (San Diego, CA, USA)	TruSight Oncology 500 [37]	523	Hybrid Capture	Illumina: sequencing by synthesis	NextSeq550Dx	No
Roche (Basel, Switzerland)	FoundationOne^®^Liquid CDx [38,39]	324	Hybrid Capture	Outsourced		N/A
Guardant Health (Moorpark, CA, USA)	Guardant360 [40]	739	Hybrid Capture	Outsourced		
SOPHIA GENETICS (Rolle, Switzerland)	MSK ACCESS powered by SOPHiA GENETICS [41]	146	Hybrid Capture	Illumina: sequencing by synthesis: Novaseq6000/NextSeq2000		Element Aviti (sequencing-by-binding chemistry)
Roche (Basel, Switzerland)	Avenio Expanded panel [42]	77	Hybrid Capture	Illumina: sequencing by synthesis	NextSeq550Dx	N/A

## 3. Liquid Biopsy: A General View for a Complementary or an Alternative Approach to a Tissue Biopsy and Potential Limitations

Despite increasing interest and a higher number of publications related to this field, the daily use of LB for NSCLC is still limited, at least in Europe [43]. In contrast, the LB is used more in North America, where the concept of «plasma first» was first set up [44,45,46,47,48]. In some regions of North America, it may take several weeks to get a medical appointment for a pulmonary endoscopy to obtain a bronchial tumor biopsy or a CT scan for a transthoracic tumor biopsy, depending on the state and the medical care organization. Thus, the delay to obtain the results of molecular tests from tissue samples can be too extensive, leading to a delay that is incompatible with the administration of an appropriate treatment, particularly a targeted therapy [45]. In addition, the cost effectiveness of blood sampling in comparison with patient hospitalization for invasive procedures is much higher. This leads to inequality in health, notably according to race and ethnicity [45,49]. Taking into consideration the economics and the trend towards the practice of an LB as a first step and, depending on the results of the molecular tests, a subsequent TB for molecular assessment from tumor samples can be proposed [50]. In France and in different European countries, patient care based on a TB is a more rapid process, which probably allows the molecular results from tissue and/or cytological samples to be obtained in a reasonable time for treatment decision-making. Therefore, the concept of “plasma first” in these countries is not the usual practice, and doing an LB at diagnosis appears to be more of a complementary approach, which can follow in a sequential manner a TB if the molecular results obtained from the latter are not informative or not available. However, some investigators suggest performing a TB and LB simultaneously with the aim to increase the chance of detecting a molecular target for personalized therapy [14,51,52,53]. This practice is quite controversial at baseline before any treatment, but is of major importance at tumor progression, notably for patients receiving TKI, since secondary resistant mechanisms can be present from different cellular clones in various metastatic sites and therefore can be rapidly detected at the same time with an LB [23,54,55,56]. Indeed, these different cellular clones of resistance cannot be assessed at a single TB site. Finally, in this clinical setting, a tissue rebiopsy can be performed in a sequential manner if the LB is not informative enough to switch to another targeted therapy [57,58,59,60].

## 4. Liquid Biopsy and/or Tissue Biopsy: International Recommendations for Best Practice and Other Algorithms

**Figure 2:**
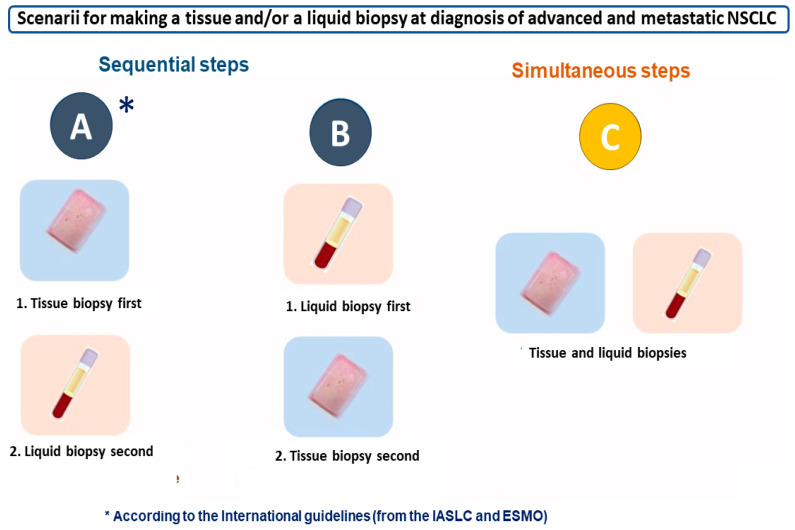
International recommendations (**A**) and possible algorithms (**B**,**C**) for tissue and liquid biopsy practice for advanced and metastatic nonsquamous nonsmall cell carcinoma at diagnosis.

Different scenarios can be developed in thoracic oncology in order to use a liquid biopsy in routine clinical practice (Figure 2). The first scenario is to follow the different international guidelines, for example, from the European Society for Medical Oncology (ESMO) and from the International Association for the Study of Lung Cancer (IASLC), which have been published for LB best practice use in advanced and metastatic NS-NSCLC [61,62]. Therefore, at baseline, in the case of a suspected advanced and metastatic NSCLC, an LB has to be performed only if the TB is not available (or not possible to be performed), or if the tissue and the cytological material are associated with an insufficient quantity and/or quality of extracted nucleic acid, and/or with an initial diagnosis of a low percentage (<10%) of tumor cells in the hematoxylin-eosin-stained tissue section, notably for molecular sequencing testing (using one gene sequencing testing and/or DNA/RNA NGS) [12,63]. An LB can be performed if the TB is scheduled with a long delay that is not compatible with optimal care [64]. Additionally, the Blood First Assay Screening Trial (BFAST) was set up to use LB first in advanced/metastatic NSCLC. It is a global, open-label, multicohort trial that evaluates the efficacy and safety of different therapies in these patients with targetable alterations identified by LB [65,66,67,68]. At tumor progression, an LB can be performed first to assess rapidly the presence of a secondary-resistant TKI mechanism, which could be drug-targetable [69]. However, a TB should be performed systematically if possible, according to the performance status of the patient and/or the availability of the biopsy site [57,59].

An alternative clinical decision is to take both a TB and LB at diagnosis and is favored by some investigators, in particular when using large panels of genes (Figure 2) [15,47,51,52,53,70]. This algorithm is debatable since all the genomic alterations that are currently druggable thanks to a targeted therapy can be in theory detectable on a TB of “good quality” (meaning with a sufficient quantity of tumor cells), and thus, an additional LB would not be helpful to detect additional targetable genomic alterations. This matched LB would then be only useful if the TB is not informative, as already mentioned above. The possibility of obtaining at the same time in the laboratory the TB and LB can save time to obtain all the results, avoiding the need to ask the patient to come back to the clinical department for blood sampling at a second time. From a practical point of view, ct-DNA preparation could be performed systematically, ready for NGS in the case of a noninformative NGS from the TB. At tumor progression, the detection of certain genomic alterations with a LB can also allow a patient to be included in a clinical trial, in particular when using NGS with a large gene panel [57].

When the results of an LB are negative, the interpretation becomes a major issue (leading to the conclusion of a “non-informative” LB). Therefore, the challenge is to be able to distinguish between a true and false negative result [71]. In this regard, identifying the ct-DNA fraction in the cf-DNA is pivotal to confirm a true negative result (within the limit of the size of the gene panel), and thus the absence of a targetable genomic alteration. Detection of the ct-DNA fraction is currently not so easy to perform in daily practice in all laboratories and needs technical expertise. One way of improving detection in routine is to perform biological testing to detect the presence of methylated genes that confirm the presence of ct-DNA. This can be performed notably by using a dPCR method or by NGS with a targeted methylation signature. False negative results can also be related to the use of low-sensitivity methods that are not able to detect genomic alterations in a very low quantity of ct-DNA. Conversely, LB can lead to false positive results. Therefore, if some *EGFR* somatic mutations are detected in an LB but not in a TB containing extracted DNA of good quantity and quality, the diagnosis of a false positive result is strong. This clinical setting should lead to the diagnosis of a germline *EGFR* mutation detected in cf-DNA (germline DNA contamination from blood cells). This diagnosis can then be confirmed with DNA extracted from circulating leukocytes (from PBMC or buffy coat). However, a false negative result with TB, due to an insufficient amount of tumor DNA must be eliminated (for example, in the case of degraded tumor DNA and/or low sensitivity of the molecular testing approach). When doing simultaneously NGS on a TB and LB, it is also possible to distinguish mutations associated with a clonal hematopoiesis of indeterminate potential (CHIP), which are only present in blood and more particularly in circulating blood cells.

The tumor mutation burden (TMB) biomarker in tissue samples but also in blood [blood TMB (bTMB)] was an initial promising predictive biomarker of immunotherapy response [72,73,74,75]. However, the bTMB is not used currently in daily practice, and the TMB of tissues is not used routinely as a predictive biomarker in France. It is noteworthy that there are usually different values between the TMB assessed in matched TB and LB, since bTMB can be overestimated thanks to some «background», notably mutations associated with the CHIP or germline mutations. Thus, ideally, the bTMB in cf-DNA should be evaluated after the assessment of the CHIP/germline mutations detected with NGS on extracted DNA from white blood cells from the same blood sampling and after bioinformatic filtering.

TB is the gold standard approach for diagnosis of NSCLC and histological subtype identification, as well as for some predictive biomarker assessment using IHC (such as immunostaining for PD-L1 and ALK), FISH (*ALK*, *ROS1*, and *MET*), and molecular biology testing (one gene sequencing and NGS methods). Moreover, the sensitivity for fusion and copy number variation detection is generally higher with a TB than a LB, particularly when the tumor fraction in cf-DNA is low (<1%) [17,51,76]. The prevalence of driver rearrangements is generally lower with a LB than a TB, but the frequency of detection can be comparable in a LB with a tumor fraction ≥1% [77]. Moreover, some of these discrepancies can be associated with the quality of ct-DNA, but also with the quantity of the ct-DNA, depending on both biological and technological issues [76,78].

**Figure 3:**
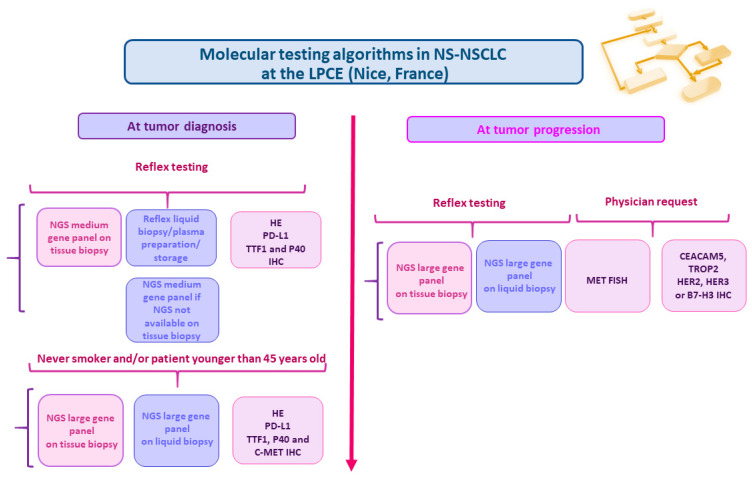
One center experience (LPCE, IHU RespirERA Nice) concerning the algorithms for use of tissue and liquid biopsies for nonsquamous nonsmall cell lung cancers (NS-NSCLC).

International guidelines for LB use in daily practice can be adapted according to the budget and reimbursement of testing, the institutions’ organization, the availability of the molecular pathology platforms, the expertise of the team, and the physicians’ requests. As an example, since 2020 in our institution (RespirERA University Hospital Institute, Nice, France), matched TB and LB are routinely performed at diagnosis (Figure 3). NGS are performed as reflex testing in advanced and metastatic NS-NSCLC with both TB and LB under the following conditions: (i) in never smoker patients, (ii) in young patients (i.e., less than 45 years old), and (iii) in case of an urgent need due to rapid tumor progression (Figure 3) [52]. For these cases, blood is centrifuged and the cf-DNA extracted from plasma is stored at −80 °C until potential future use, notably if the NGS performed on a matched TB is not informative. If NGS is not performed with extracted cf-DNA, the material is kept in the BB-0033-00025 biobank, which is ISO 20,387 certified [79], for further use (notably for inclusion into research projects, including those dedicated to benchmarking of different NGS panels). An LB is systematically performed at tumor progression, notably in patients receiving targeted therapy (Figure 3). In this context, a tissue rebiopsy ideally oriented on the site of tumor progression should be performed in the absence of clinical contraindication. This allows rapid diagnosis of a possible transformation of a histological type (in small cell carcinoma or in squamous cell carcinoma) and, in our institution, to perform systematically a *MET* FISH at tumor progression under TKIs.

The selection of the NGS panel for use with an LB varies according to the algorithm, the institution, the organization, and the sequencing practice on site, the budget, and the expertise of the team for bioinformatic assessment (Table 1). Therefore, NGS panels have a small number of genes (from 11 to 50) up to several hundred genes (over 500) (Table 1). The same panels and sequencing technologies can be used for both an LB and TB [69,77]. Globally, the large panels used for LB need to be run on certain sequencers, in particular the Novaseq Instrument (Illumina, San Diego, USA), while other smaller NGS panels can be analyzed on other sequencers according to the approach used, such as the GENEXUS and the S5 Oncomine instruments (Thermo-Fisher Scientist, Waltham, USA) and other instruments from Illumina (Next Seq 550, Next Seq 2000, Mi Seq) and more recently from MGI Tech Co (Shenzhen, China) and Aviti System by Element Biosciences (San Diego, CA, USA). To run the LB, two strategies can be setup: (i) a systematically outsourced blood sample to be analyzed, notably on commercially available platforms, or, (ii) an internalized analysis on site.

**Figure 4:**
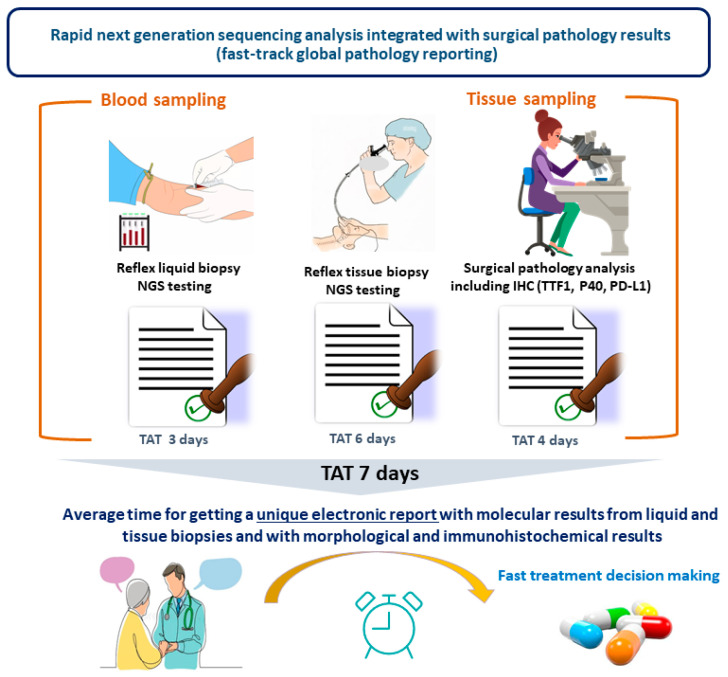
Turnaround time (TAT) optimization to obtain at the same time the report for both the pathology and molecular results.

At the IHU RespirERA, different NGS panels are used: (i) a small (12 genes) and a medium (50 genes) panel at tumor diagnosis both with a TB and LB; (ii) a large panel (324 or 670 genes) with a TB systematically at tumor progression or at diagnosis in never smokers and young patients; (iii) systematically a large panel (170 genes) with an LB at tumor progression (Figure 3). In this context, it is challenging to integrate all the results obtained from a TB and LB in the same report, including the pathological data (with the different IHC results). Therefore, the development of new sequencing technologies (such as the so-called “ultra-fast” NGS) and the improvement of automation of the preanalytical and analytical phases has led more and more to a report containing all the information at the same time for optimal treatment decision-making [80,81,82] (Figure 4). Moreover, NGS reflex testing is certainly an important option to save time for optimal treatment decision-making [83]. In our institution, both NGS with a TB and LB are performed as reflex testing. The practice of this reflex testing is guided by the adherence to a protocol, defined and agreed to by the multidisciplinary team, in accordance with local circumstances and institutional authorization. Where possible, comprehensive reflex biomarker testing, via NGS and IHC, is performed for all patients diagnosed with NSCLC (including squamous cell lung carcinoma), and irrespective of the stage of the disease. In our institution, we were able to set up reflex NGS from both TB and LB in NSCLC thanks to a collaborative project with the physicians, the Nice University Hospital, the FHU OncoAge, and more recently with the IHU RespirERA, all participating in the cost associated with these tests. Moreover, part of the cost is funded through state resources coming from the budget of the French government. Thus, since 2020, more than 500 NSCLC patients can be tested at diagnosis each year using a medium gene panel. Since currently it is certainly mandatory to look for the status of 9 genes (*EGFR, BRAF, ALK, ROS1, MET, RET, HER2, KRAS, and NTRK*) in NS-NSCLC, it would also be possible to use a small NGS panel. However, we decided to use a larger panel of 50 genes at tumor diagnosis in order to have a larger molecular portrait of the tumor, including information on the main exons of *TP53* (since *TP53* mutations may have an impact soon on treatment decision-making), but also on other potential therapeutic targets associated with genomic alteration on different genes such as *NRG1*, *PI3KA,* and other genes. Moreover, mutations on some genes, such as *STK11* and *KEAP1,* can have an impact on the treatment efficiency.

## 5. Perspectives and Conclusions

The daily use of an LB for lung cancer patients is currently performed for cases of advanced and metastatic NS-NSCLC [58]. In this regard, until now, the international guidelines for use of an LB in these patients have not concerned other clinical situations. As an example, plasma-based *EGFR* mutation detection post-treatment initiation can be used as a predictive marker for outcome in patients with *EGFR*-mutant NSCLC receiving first-line treatment [84]. Other possibilities might be developed in the next few months or years, such as including molecular testing for squamous cell lung carcinoma and small cell lung carcinoma [85,86,87]. The development of ultrasensitive molecular testing may also be helpful to integrate the indication of an LB into early-stage NSCLC before surgery, aiming to better select patients who can receive neoadjuvant immunotherapy or adjuvant *EGFR* TKI therapy [88,89]. One important perspective is to use a LB to assess the minimal residual disease (MRD) after completing surgically resected NSCLC [12,90,91,92,93,94]. In the presence or in the absence of MRD, patients receiving an adjuvant therapy could receive it during follow-up treatment escalation or, conversely, treatment deescalation [92,95]. However, different parameters (including which molecular test to be select, which cutoff of ct-DNA, at what time (s) postsurgical resection to do blood sampling, how to master the cost and the reimbursement of testing, notably in a monitoring perspective, and how to integrate the increasing workload in daily practice) need to be mastered before using MRD testing in routine [96].

**Figure 5:**
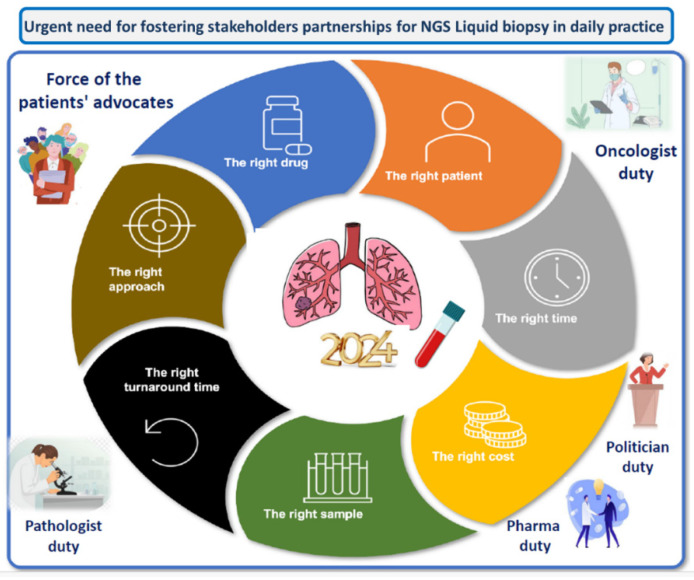
Different stakeholders who can provide an optimization of next generation sequencing liquid biopsy implementation in routine clinical practice.

Optimal routine use of LB for lung cancer patients is facing different challenges. One major issue of using a LB in daily practice for lung cancer patients is certainly the cost and the absence of reimbursement, notably when using NGS, and the perspective of this cost when monitoring the disease in targeted treated NSCLC patients and the repetition of the blood sampling. Moreover, LB could soon be used to monitor all lung cancer histological subtypes, such as small cell lung carcinoma, and to detect new therapeutic targets thanks to the assessment of different molecular biomarkers [86]. Finally, besides using an LB for the detection of genomic alterations associated with potential targeted therapies, LB could be used more and more when administering immunotherapy to evaluate the therapeutic responsiveness and new resistant mechanisms [97,98,99]. As mentioned above, one major goal is to integrate the practice of doing LB into the daily standard of patient care, which is economically quite difficult in terms of sustainability, notably since the reimbursement of these blood tests is not certain in most countries [100]. Therefore, the different stakeholders, including the politicians, the pharmaceutical and biotechnological companies, but also the advocates of patients, the physicians, and the molecular pathologists, need to work together to rapidly find solutions for molecular testing with TB and extend the use of LB in all countries [100] (Figure 5). One major possibility could be to have a strong commitment of different pharmacology companies at the national or at the international levels for participating in the budget associated with NGS LB. These companies could be organized in large consortia working together with different official authorities (such as the ministries of health or the national cancer institutes) in order to regulate the cost and the implementation of LB in the health care system.

**Figure 6:**
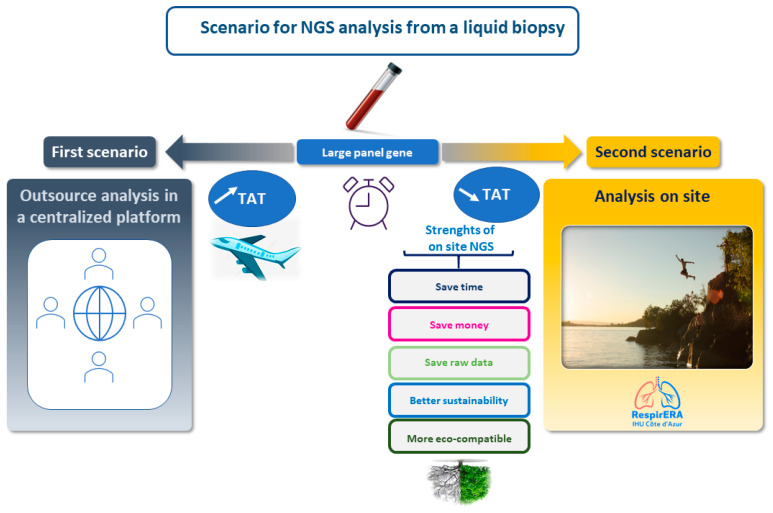
Different scenarii for NGS with liquid biopsies for patient care.

The accessibility to performing an analysis with a LB for lung cancer patients, notably in Europe, is still very variable, notably when using the NGS approach, which represents a major bottleneck for the equity of care in oncology [101,102,103]. This is also at least partially linked to some relative weaknesses of the availability of technological platform infrastructures on site, leading to sending away the blood samples and to outsource the sequencing on commercially available platforms [such as Foundation Medicine (Cambridge, MA, USA) and Guardant Health (Palo Alto, CA, USA) or in other large academic platforms [such as the recently program launched in France by the Institut Gustave Roussy [104,105,106,107]] located far away from the different local institutions (where the patients are treated). Therefore, LB “democratization” and the dissemination of blood sample use for genomic alteration assessment should be associated with the possibility to perform the analyses in house/onsite in any hospital (Figure 6).

This latter option is, from our personal point of view, an urgent need, and offers several advantages: (i) a shorter TAT to obtain the report, (ii) lower costs, (iii) complete mastering of the raw data, and the opportunity for a biobank to use the leftover nucleic acid for immediate reanalysis or ancillary studies, (iv) an optimization of the «green vision» concerning the different steps of the workload, including a decrease in sample transportation from one site to another (notably by plane) (Figure 6). The increase in the number of therapeutic targets, and also of the different clinical trials in thoracic oncology, has progressively led to more frequent use of large panels of genes with several hundred genes, so-called comprehensive genomic profiling (CGP) panels. When using LB, these large panels need to be run on specific and costly instruments such as the Novaseq 6000 (Illumina) to be able to deeply sequence the low amount of nucleic acid. The usefulness of these CGP for LB is of great interest at tumor progression for patients on targeted therapies, since the resistance mechanisms are more and more complex and since there are different options for patient inclusion into clinical trials, notably into a phase I trial [107,108]. There is an urgent need to integrate complementary tests such as liquid and tissue data into the same report at the same time to provide the entire biomarker landscape to the physician [109]. In this context, it is of strong interest to follow different international guidelines and recommendations for reporting both tissue and liquid biopsy NGS results, together with different IHC data [not only concerning PD-L1 expression in tumor cells, but also the expression of other soon-to-be developed biomarkers, notably different proteins targetable by ADCs, such as c-MET] (Figure 4) [109]. Considering the use of these large panels of genes both with TB and LB, it will be important in the future, to facilitate treatment, to use soft reports with links to big data such as large-scale genomic/transcriptomic/proteomic databases. Finally, these reports have to integrate the impact of comutations on targeted therapies and immunotherapies and the possible primary and secondary resistance mechanisms [25]. However, thanks to this increase in the complexity of interpretation of genomic alterations, it is now mandatory to provide an easier way for the physician to use guidelines for interpretation of the results [110,111]. Finally, it is noteworthy that despite the potential that NGS, in particular with LB, offers for diagnosis and adapting personalized therapy, only a few patients benefit from this approach, at least in France and many countries in Europe, because of the failure of the health care system and policymakers to implement them in daily practice. Thus, it is urgent to convince different stakeholders, at least financial authorities, that LB NGS should now be performed not only in clinical trials, but also routinely for clinical care of NSCLC patients [112]. One point to highlight here is that as for TB NGS, when doing LB NGS both in daily practice but also in clinical trials, the physicians must obtain the signed consent form from patients. However, this process is burdensome since consent forms are not easily readable for patients, and the time necessary to complete the consent process can be a barrier to participation [113]. Thus, the consent process associated with the LB NGS obtention needs to be improved to make it more responsive to the needs of participants. One major issue is certainly to be able to make an LB in low-funding countries, low-resource institutions, and in the absence of national reimbursements. Narrowing the huge existing disparities in incorporating NGS LB into healthcare systems urgently requires different initiatives. The optimization of education, the establishment of standardized procedures, the allocation of resources, and infrastructure investment are some of the current challenges for NGS LB implementation in certain countries. Moreover, the promotion of strong collaboration and support, among distinct groups of stakeholders, including pharmacology companies, is pivotal to helping low-funding countries to be able to develop LB use. Probably LB use in thoracic oncology daily practice could be more restrictive in some countries and thus limited to tumor progression only or in case of not any possibility to have access to a tissue biopsy.

Finally, different concepts are currently emerging in oncology, notably the possibility of considering a solid tumor with more molecular classifications than organ-based classifications [114]. This can provide added value for better optimization of patient inclusion into many clinical trials (such as for “basket trials”) using targeted therapies [115]. However, the perspective for NSCLC patients is to integrate massive data from sequencing using large panels of genes both with tissue and blood samples, not only at tumor progression but also at tumor diagnosis, in addition to clinical and pathological data. This has led us and others to still believe that it is important to keep using organ-based classifications in oncology, at least for the thoracic oncologist [116,117]. Finally, in this context, we believe that the increased use of LB for solid tumors, and thus in lung cancer patients, should lead us to maintain the identity of the solid tumor-based visibility in oncology so as to maintain a strong link and communication between the physicians and the clinical and molecular pathologists.
